# Global Food Safety—International Consumers’ Rights?

**DOI:** 10.3390/foods2040460

**Published:** 2013-10-11

**Authors:** Malik Altaf Hussain

**Affiliations:** Department of Wine, Food and Molecular Biosciences, Lincoln University, Lincoln 7647, Canterbury, New Zealand; E-Mail: Malik.Hussain@lincoln.ac.nz; Tel.: +64-3-325-3838; Fax: +64-3-325-3851

Your submissions to this Special Issue “Food Microbiology and Safety” of *Foods*—a new open access journal—are welcome. We understand there are no foodborne illness-free zones in the world. Therefore, a proper understanding of foodborne pathogens and the factors that impact their growth, survival and pathogenesis would equip us with tools to ensure global food safety. This Special Issue publishes articles on different aspects of food microbiology and safety.

Globalization of the food trade has complicated foodborne hazards. On the one hand, international food trade laws are not yet defined and coordinated. On the other hand, different government policies and regulations are designed to boost the country’s economy through large volumes of food exports. This trade priority generally favors the food industries rather than their consumers. This is the reason why little has been done so far to address some of the root issues in global food safety, *i.e.*, standards for the global food chains/producers and international consumers’ rights. Many developing countries are becoming major suppliers of global food products while still facing safe food handling issues, poor sanitation and potable water supply systems. Moreover, rapid urbanization in these countries is putting pressure on commercial food production and processing sectors, which may lead to compromises in food safety practices to meet the demand from domestic and international markets.

Microbiological food safety issues are complex and diverse. A number of factors are serving as catalysts for foodborne outbreaks including large-scale production practices, complex food supply chains and globalized distribution networks [1]. Foodborne outbreaks involving microbial pathogens may lead to health risks and financial losses. Overall economic costs from foodborne outbreaks include loss of individual income, loss of productivity, cost of health care, cost to investigate an incident, cost of product wastage and loss of business income and sales. We need to understand that consumers demand safe food products and this is a great incentive for the companies to produce safe foods. There is no doubt that the implementation and maintenance of food safety systems would add extra costs to companies. It has been demonstrated that the cost of handling food safety outbreaks are much higher than the cost of preventing them [2]. Therefore, more practical measures are needed to reduce the number of food safety incidents. An opinion article on the “Economic impact of food safety outbreaks on food businesses” in this Special Issue will discuss the implications of food safety incidents.

Around the world, complex changes are happening in the way people buy and consume foods. In addition, some people are leaving their traditional eating habits to adapt to the global trend of fast foods and processed foods. In other places, economic growth is causing a shift to being able to afford more dairy and meat products. Elsewhere people are abandoning ready-to-eat foods for more fresh produce. These transitions are the driving force behind the growing international trade of new food items and the creation of new food safety scares [3]. Hundreds of scientists are studying food safety problems and solutions for these dynamic international food supply chains. Their results will improve our knowledge to develop integrated systems to minimize food safety issues and allow us to stay ahead of emerging scandals.
